# Tiao-Jing-Cu-Yun Acupuncture combined with estradiol in the treatment of patients with thin endometrium undergoing hormone replacement therapy for frozen embryo transfer: a randomized controlled trial

**DOI:** 10.3389/fmed.2026.1811023

**Published:** 2026-05-21

**Authors:** Junmin Li, Xiaona Yang, Yichun Guan

**Affiliations:** Reproductive Health Hospital, The Third Affiliated Hospital of Zhengzhou University, Zhengzhou, China

**Keywords:** acupuncture, endometrial blood flow, endometrial receptivity, frozen–thawed embryo transfer, randomized controlled trial

## Abstract

**Objective:**

To investigate the effect of Tiao-Jing-Cu-Yun Acupuncture on pregnancy outcomes in infertility patients with thin endometrium undergoing artificial cycle frozen–thawed embryo transfer (AC-FET).

**Methods:**

A prospective, assessor-blinded randomized controlled design was employed. A total of 92 infertility patients with endometrial thickness <7 mm undergoing AC-FET at our hospital were enrolled and randomly assigned to two groups: the control group (*n* = 45) received oral estradiol valerate-based hormone replacement therapy combined with sham acupuncture; the Intervention Group (*n* = 47) received the same HRT regimen combined with Tiao-Jing-Cu-Yun Acupuncture administered every other day starting from menstrual cycle day 2–3 until the day before embryo transfer. The primary outcome measure was the clinical pregnancy rate. Secondary outcomes included the embryo implantation rate, early miscarriage rate, and changes in endometrial parameters assessed via transvaginal ultrasound before and after treatment, including endometrial thickness, volume, pattern classification, subendometrial blood flow grading, and hemodynamic parameters.

**Results:**

Baseline characteristics were comparable between the two groups. After treatment, the Intervention Group showed a significantly greater increase in progesterone (P) level, endometrial thickness and volume, and a more pronounced decrease in endometrial resistance index (RI) and pulsatility index (PI) compared to the control group (all *p* < 0.05). Ultrasound assessment revealed that the proportion of patients achieving Pattern A endometrium and Grade III subendometrial blood flow after treatment was significantly higher in the Intervention Group than in the control group (*p* < 0.05). Regarding pregnancy outcomes, the clinical pregnancy rate in the Intervention Group (55.32%) was significantly higher than that in the control group (31.11%), and the embryo implantation rate was also significantly increased (all *p* < 0.05). There was no statistically significant difference in the early miscarriage rate between the two groups (*p* > 0.05).

**Conclusion:**

The combination of Tiao-Jing-Cu-Yun Acupuncture with standard hormonal therapy can effectively improve endometrial receptivity in patients with thin endometrium and significantly enhance the clinical pregnancy success rate of frozen–thawed embryo transfer.

**Clinical Trial Registration:**

https://itmctr.ccebtcm.org.cn/, International Traditional Medicine Clinical Registry (ITMCTR), unique identifier: ITMCTR2026000585.

## Introduction

In recent years, the incidence of infertility has shown an increasing trend both domestically and internationally. The incidence of infertility in China is approximately 17.5–18.5%, making it a common gynecological condition (1, 2). The causes of infertility are complex, among which thin endometrium and decreased endometrial receptivity are significant contributing factors (3–5). An endometrium of adequate thickness provides a favorable internal environment for conception. When the endometrium reaches a certain thickness, glandular secretion becomes vigorous, progesterone levels are higher, and endometrial receptivity is greater (6, 7). Therefore, repairing the damaged endometrium, altering endometrial morphology and local blood flow, improving endometrial receptivity, and promoting endometrial growth to meet the requirements for pregnancy are urgent issues for infertility patients. In all assisted reproductive technologies, especially during frozen–thawed embryo transfer (FET) cycles, hormone replacement therapy (HRT) is commonly used for endometrial preparation (8). However, some women respond poorly to conventional estrogen therapy, persistently presenting with thin endometrium and/or poor endometrial morphology, a condition referred to as “thin endometrium.” This directly leads to reduced embryo implantation rates and increased cycle cancellation rates, representing a major bottleneck in current clinical practice (9). For thin endometrium, current clinical approaches mainly include increasing estrogen dosage or duration, and adding medications such as aspirin (9). Among these, oral or vaginal administration of estradiol valerate (EV) serves as the foundation and mainstream of HRT regimens. Although this therapy can effectively promote endometrial growth in most patients, its efficacy varies individually. Some patients show suboptimal responses, and long-term or high-dose use may carry potential side effects such as increased thrombotic risk and breast discomfort (10). Hence, exploring safe, effective, and individualized adjuvant therapies to improve endometrial receptivity is of great significance for enhancing the success rates of assisted reproduction.

Against this backdrop, traditional Chinese medicine (TCM) intervention strategies demonstrate unique potential. As a characteristic external treatment modality based on TCM theory and meridian doctrine, the Tiao-Jing-Cu-Yun Acupuncture has a long history in regulating gynecological disorders. Furthermore, applying this acupuncture method to treat thin endometrium not only avoids the toxic side effects associated with medications but also offers strong therapeutic effects and rapid efficacy, fully reflecting the distinctive features and advantages of TCM in treating infertility. Therefore, this randomized controlled trial (RCT) aims to systematically evaluate the effects of combining regulated menstruation and fertility-promoting acupuncture with estradiol valerate in hormone replacement therapy frozen–thawed embryo transfer cycles on endometrial thickness, morphology, clinical pregnancy rate, and live birth rate in women with thin endometrium.

## Materials and methods

### Sample size calculation

The sample size for this study was calculated based on the primary outcome measure. Assume that the clinical pregnancy rate in the estradiol valerate plus sham acupuncture group (Control group) is 32% (11), we hypothesized that the combination with Tiao-Jing-Cu-Yun Acupuncture (Intervention Group) could increase this rate to 60%. This trial is designed as a superiority design. With an *α* level of 0.05, a power (1−*β*) of 80%, the calculation indicated a requirement of at least 37 patients per group. Accounting for an anticipated dropout rate of 10%, we plan to recruit a minimum of 41 patients per group, resulting in a total target sample size of 82 participants.

### Study design

**Figure 1:**
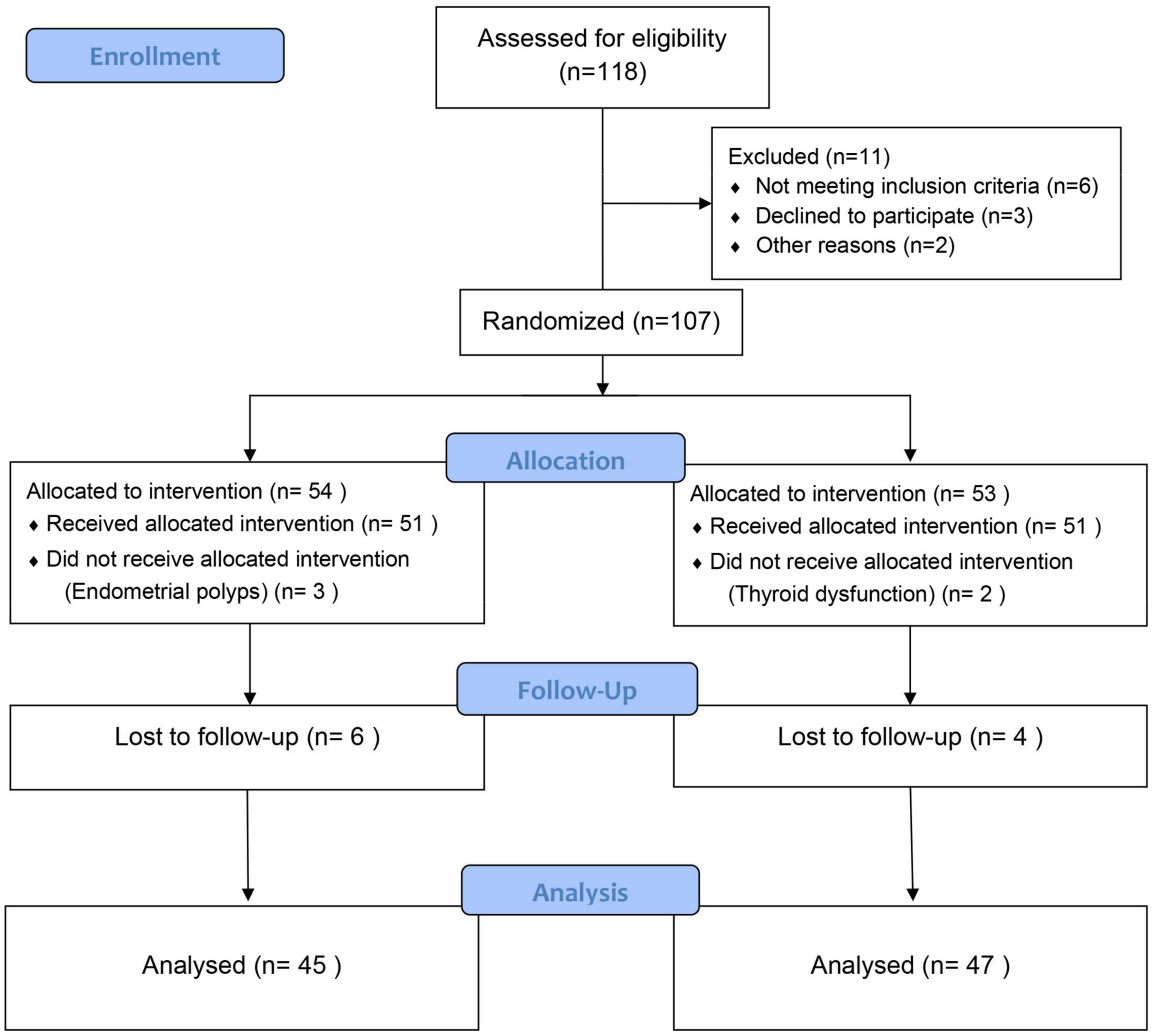
Study flow diagram.

This study is a prospective, assessor-blinded RCT. Between January 2023 and September 2024, a total of 118 infertility patients with thin endometrium undergoing artificial cycle frozen–thawed embryo transfer (AC-FET) (with day-3 cleavage-stage embryos transferred) were enrolled from the Reproductive Medicine Department of the Third Affiliated Hospital of Zhengzhou University. During the follow-up period, 6 cases withdrew from the control group and 4 cases withdrew from the experimental group, resulting in a total of 92 patients who completed the full follow-up and were included in the analysis. Patients were randomized in a 1:1 ratio using a complete random number table into two groups: the Control group (treated with Progynova combined with sham acupuncture, *n* = 45) and the Intervention Group (treated with Progynova combined with Tiao-Jing-Cu-Yun Acupuncture, *n* = 47). The sample size met the minimum requirement. The patient screening and grouping process is detailed in Figure 1. All patients were followed up for 3 months. The trial was conducted in accordance with the Declaration of Helsinki, informed consent was obtained from all participants, and the study protocol was formally approved by the hospital’s Ethics Committee (Approval No.: 2023-366-01). This study was registered with the International Traditional Medicine Clinical Registry (https://itmctr.ccebtcm.org.cn/), under registration number: ITMCTR2026000585.

### Blinding and implementation

This study employed a assessor-blinded design. Due to the specific nature of acupuncture treatment, complete double-blinding was not feasible for the acupuncturists and the patients. However, to minimize measurement and assessment bias, blinding was implemented for the outcome assessors and data analysts. All ultrasound measurements were performed independently by dedicated physicians who were blinded to group allocation. The determination of pregnancy outcomes was also conducted by clinical physicians blinded to group assignment, based on predefined criteria. During the data analysis phase, statisticians had access only to anonymized group codes; unblinding occurred only after the analysis was completed.

### Patient population

Inclusion criteria were as follows: (1) Female age between 18 and 40 years with fertility desire; (2) Normal uterine morphology confirmed by hysteroscopy (HSC) or hysterosalpingography (HSG); (3) Endometrial thickness <7 mm confirmed during the proliferative phase of the current cycle prior to randomization; (4) Undergoing frozen–thawed embryo transfer (FET); (5) Body mass index (BMI) between 18 and 28 kg/m^2^. Exclusion criteria were as follows: (1) Patients with endocrine disorders such as thyroid dysfunction, ovulatory dysfunction, or hyperprolactinemia; (2) Patients with organic lesions such as uterine fibroids, endometrial polyps, endometriosis, or ovarian cysts; (3) Patients with mental disorders, cognitive impairment, or communication barriers; (4) Chromosomal abnormalities in either spouse; (5) Recurrent implantation failure (RIF) (defined as ≥2 previous failed transfers). Withdrawal and Elimination Criteria included: (1) Extremely poor patient compliance, violating the study protocol requirements; (2) Poor quality of data recording, with incomplete or inaccurate information; (3) Loss of contact with the patient.

## Methods

### Treatment protocol

Intervention Group received standard hormone replacement therapy combined with acupuncture therapy for regulating menstruation and promoting fertility. Patients began oral administration of estradiol valerate tablets (Progynova, Bayer Healthcare Co., Ltd., National Medicine Approval No. J20171038, 1 mg/tablet) on day 3 of the menstrual cycle, with a starting dose of 4 mg daily administered in two divided doses. Endometrial growth was monitored regularly via transvaginal ultrasound. Progesterone was added for endometrial transformation (progesterone injection 60 mg/day intramuscularly) when the endometrial thickness reached or exceeded 7 mm. A high-quality D3 embryo (≥7 cells, grade II) was transferred on the third day after transformation. Luteal phase support was continued until the pregnancy test day. For acupuncture treatment, disposable sterile acupuncture needles (Hua Tuo brand) of sizes 0.25 × 25 mm, 0.25 × 40 mm, and 0.25 × 75 mm were used. Acupoints included abdominal points [Zhongwan (CV12), Tianshu (ST25), Guanyuan (CV4), Zigong (EX-CA1), Dahe (KI12)], lower limb points [Zusanli (ST36), Sanyinjiao (SP6), Taixi (KI3), Taichong (LR3)], back points [Shenshu (BL23), Ciliao (BL32)), and head points (Benshen (GB13), Shenting (GV24), Baihui (GV20)]. Treatment consisted of two abdominal sessions and one back session per week, alternating. During the procedure, abdominal points were perpendicularly needled 1–1.5 cun, aiming for a sensation radiating to the lower abdomen. For the back points, Shenshu (BL23) was needled 0.5–1 cun perpendicularly, and Ciliao (BL32) was deeply needled 3 cun into the posterior sacral foramen. Limb points were needled perpendicularly until deqi was obtained. Head points were subcutaneously needled approximately 1 cun. Even reinforcing-reducing manipulation was applied after deqi, and needles were retained for 20 min. Treatment started on menstrual days 2–3, was administered every other day, and continued until the day before embryo transfer.

**Figure 2:**
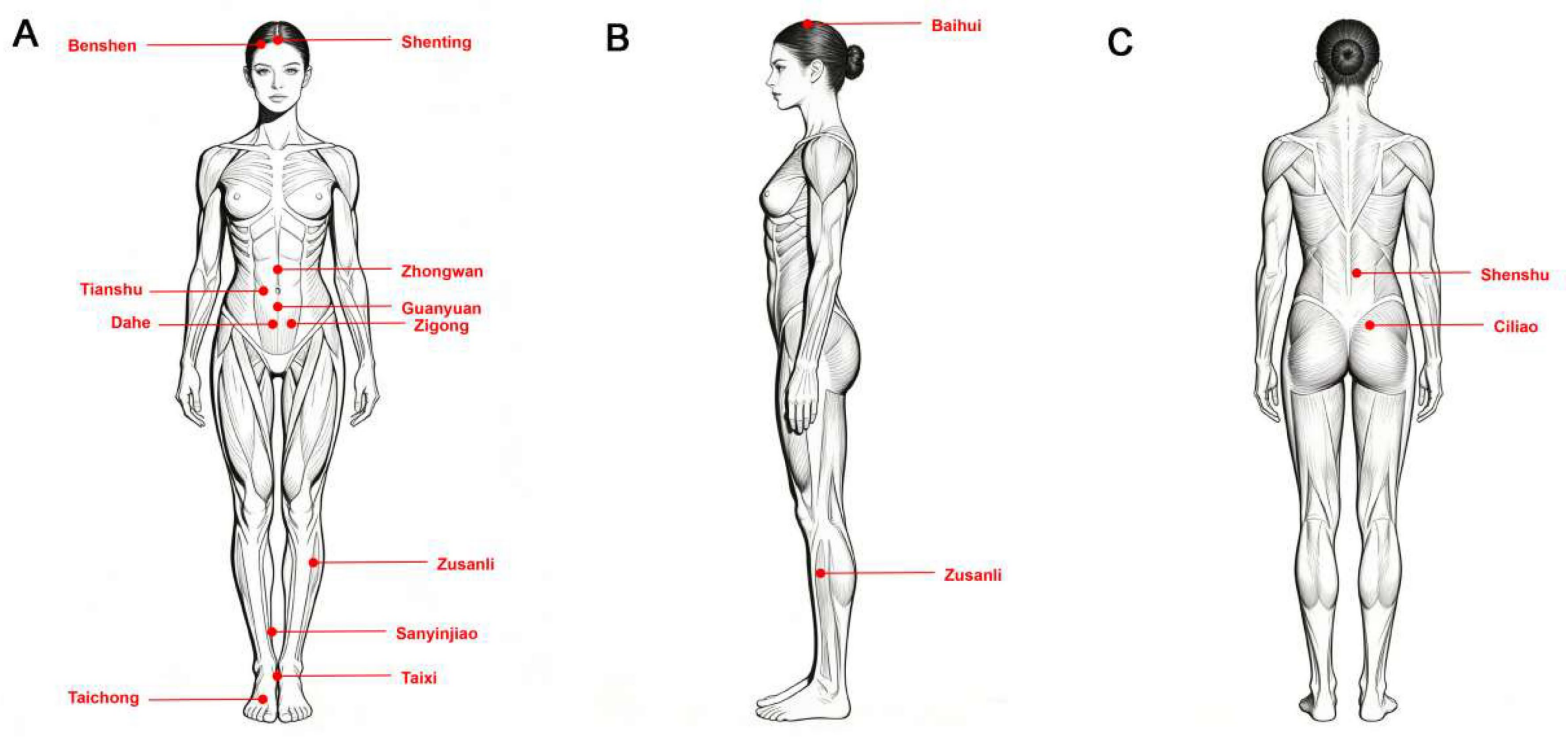
Therapeutic acupoint diagram. **(A)** Frontal acupoint diagram; **(B)** side diagram of acupoints; **(C)** back acupoint diagram. Baihui: located at the midpoint of the vertex of the head; Benshen: located 0.5 cun superior to the anterior hairline and 3 cun lateral to the midline of the head; Zigong: located 4 cun inferior to the umbilicus on the anterior midline, and 3 cun lateral to the midline; Dahe: located 4 cun inferior to the umbilicus and 0.5 cun lateral to the anterior midline; Sanyinjiao: Located 3 cun superior to the tip of the medial malleolus; Taixi: located at the midpoint between the medial malleolus and the Achilles tendon; Shenshu: located 0.5 cun lateral to the lower border of the spinous process of the second lumbar vertebra; Zhongwan: located in the upper abdomen, on the anterior midline, 4 cun superior to the center of the umbilicus; Tianshu: located in the abdomen, level with the center of the umbilicus, 2 cun lateral to the anterior midline; Guanyuan: located in the lower abdomen, on the anterior midline, 3 cun inferior to the center of the umbilicus; Zusanli: located on the lateral aspect of the lower leg, 3 cun inferior to Dubi, one finger-width lateral to the anterior crest of the tibia; Taichong: located on the dorsum of the foot, in the depression distal to the junction of the bases of the first and second metatarsal bones; Ciliao: located in the sacral region, directly over the second posterior sacral foramen; Shenting: Located on the head, 0.5 cun directly above the midpoint of the anterior hairline.

Control Group received standard hormone replacement therapy combined with sham acupuncture intervention. This group of patients followed the exact same hormone replacement therapy protocol as the Intervention Group. The sham acupuncture procedure utilized disposable placebo blunt-tip needles (Suzhou Medical Supplies Factory Co., Ltd.) and matching guide tubes designed to resemble real needles. The acupoint locations were identical to those in the Intervention Group. During operation, the placebo needle was gently tapped against the skin at a 90° angle, simulating the sensation of needle insertion through the sound of the guide tube’s retraction, while its blunt tip ensured the skin was not punctured. The practitioner then simulated needle manipulation techniques performed after “deqi,” including lifting and thrusting (amplitude approximately 0.5 cm) and rotating (angle controlled within 180°) the needle handle, followed by needle retention for 20 min. This process did not produce actual acupuncture stimulation or deqi sensation and was primarily intended for blinding purposes. The treatment frequency and duration were identical to those in the Intervention Group. All procedures were performed by operators who received unified training and followed a standardized operation manual to ensure intervention consistency. The diagram of acupuncture points applied is shown in Figure 2.

### Embryo thawing and transfer

All day-3 cleavage-stage embryos in this study were cryopreserved using the vitrification technique. Following the basic principles described by Kuwayama (12) and utilizing a protocol optimized for cleavage-stage embryos at our center, the procedure was completed using the Cryotop carrier. Specifically, embryos were placed in an equilibration solution containing 7.5% ethylene glycol (EG) and 7.5% dimethyl sulfoxide (DMSO) (Kitazato Vitrification Kit, Shizuoka, Japan) at room temperature for 5–10 min. They were then transferred to a vitrification solution containing 15% EG, 15% DMSO, and 0.5 mol/L sucrose for 20–30 s. Each embryo was loaded individually onto the polypropylene strip of the Cryotop with a minimal carrier fluid volume (<0.1 μL), rapidly plunged into liquid nitrogen, capped with a protective cover, and stored in a −196 °C liquid nitrogen tank. The thawing process followed the aforementioned protocol (12). The Cryotop carrier was immersed in a 37 °C 1 mol/L sucrose thawing solution (Kitazato Thawing Kit) for 30–45 s. Subsequently, the embryo was transferred to a 0.5 mol/L sucrose dilution solution for 2 min, followed by three washes in a sucrose-free culture medium, each lasting 1–2 min. Surviving day-3 cleavage-stage embryos were cultured for 1–2 h before uterine transfer.

### Outcome measures

#### Measurement of endometrial thickness, volume, and blood flow parameters

Endometrial thickness, volume, and blood flow parameters were measured before treatment and on the day before embryo transfer (after treatment) using a color Doppler ultrasound system (GE LOGIQ S6, USA) equipped with a transvaginal volume probe (IC5-9-D, frequency 5–9 MHz). With the patient in a resting state and with an empty bladder, endometrial thickness was measured in the mid-sagittal plane of the uterus and defined as the maximum vertical distance between the endometrial–myometrial junctions of the anterior and posterior uterine walls. Three consecutive measurements were taken by the same observer and averaged. Endometrial volume was obtained by three-dimensional ultrasound with automated volume calculation (VOCAL) after manual delineation of the endometrial boundaries. For hemodynamic assessment, the sample box was placed over the endometrial and subendometrial regions in color Doppler mode; at the site of richest flow, pulsed-wave Doppler was used to acquire the spectrum, and the pulsatility index (PI) and resistance index (RI) were recorded (13). All ultrasound examinations were performed by the same physician with more than 5 years of experience in reproductive ultrasound, under quiet conditions and with the patient’s bladder empty.

#### Endometrial pattern and subendometrial blood flow grading

Before and after treatment, endometrial patterns were classified under ultrasound according to their morphology. The criteria were: Pattern A: triple-line or multilayered; Pattern B: weak triple-line; Pattern C: homogeneous hyperechoic (14). Subendometrial blood flow was graded using the Applebaum grading system (15): Grade I: vessels penetrating the outer hypoechoic layer of the endometrium but not reaching the hyperechoic outer edge; Grade II: vessels penetrating the hyperechoic outer edge of the endometrium but not entering the hypoechoic area; Grade III: vessels entering the hypoechoic area of the endometrium. All classifications and gradings were independently assessed by two physicians blinded to group allocation. Any discrepancies were adjudicated by a third senior physician to reach a final determination.

#### Hormone levels

Serum estradiol (E₂) and progesterone (P) levels were measured using a Roche Cobas e601 fully automated electrochemiluminescence immunoassay system with its matched reagent kits. All blood samples were collected in the morning under fasting conditions at two time points: before treatment and on the day before embryo transfer (after treatment). After collection, samples were centrifuged to separate serum, which was then stored at −80 °C until analysis. The entire detection process adhered to strict quality control protocols.

#### Pregnancy outcomes and assessment criteria

Biochemical pregnancy: Serum β-hCG level ≥25 mU/L measured 14 days after embryo transfer, with no gestational sac visualized. Clinical pregnancy: Observation of ≥1 gestational sac(s) via transvaginal ultrasound. Implantation rate: The number of gestational sacs was determined by ultrasound 28–30 days post-transfer. Implantation rate (%) = (Total number of gestational sacs observed on ultrasound)/(Total number of embryos transferred) × 100%. Clinical pregnancy rate: The presence of an intrauterine gestational sac was confirmed by ultrasound examination 28–35 days after embryo transfer. Clinical pregnancy rate = (Number of clinical pregnancy cycles/Number of transfer cycles) × 100%. Early miscarriage rate: Embryo demise or spontaneous abortion occurring before 12 weeks of gestation was considered an early miscarriage. Early miscarriage rate = (Number of miscarriages within 12 weeks of clinical pregnancy) / (Total number of clinical pregnancies) × 100%.

### Outcomes

The primary endpoint of this study was the clinical pregnancy rate, defined as a pregnancy with fetal cardiac activity confirmed and progressing beyond 12 weeks of gestation. Secondary endpoints included the implantation rate, early miscarriage rate, endometrial thickness, pattern, blood flow parameters, etc. Furthermore, the implantation rate was defined as the percentage of transferred embryos that developed to the stage of documented fetal heartbeat on ultrasound. The miscarriage rate was calculated as the percentage of spontaneous abortions before 12 weeks of gestation relative to the number of transfer cycles. A biochemical pregnancy was defined as evidence of conception based solely on serum biochemical data without ultrasound evidence of a gestational sac.

### Statistical analysis

Data analysis was performed using SPSS software (version 27.0). Categorical data were compared between groups using the chi-square (*χ^2^*) test. For continuous data, if normally distributed, results are presented as mean ± standard deviation (*x* ± *s*), and inter-group comparisons were made using the independent samples *t*-test, while intra-group before-and-after comparisons employed the paired samples t-test. For non-normally distributed data, results are described as median M (Q_25_, Q_75_), with inter-group comparisons conducted using the Mann–Whitney *U* test and intra-group comparisons using the Wilcoxon signed-rank test. A *p*-value <0.05 was considered statistically significant.

## Results

### General information of the two groups of patients

The baseline characteristics of the patients are detailed in Table 1. There were no statistically significant differences in general data between the two groups (all *p* > 0.05).

**Table 1:** Comparison of general information of the two groups of patients.

Parameter	Control group (*n* = 45)	Intervention group (*n* = 47)	*Z/t/χ* ^2^	*p*
Age (years, $\bar{x}\pm s$ )	31.67 ± 4.79	30.68 ± 4.39	1.030	0.306
BMI (kg/m^2^, $\bar{x}\pm s$ )	23.15 ± 1.78	23.79 ± 1.66	−1.801	0.075
Duration of infertility [years, M (Q_45_, Q_75_)]	2.00 (2.00, 3.00)	3.00 (2.00, 3.00)	−1.052	0.293
Type of infertility (*n*, %)			0.320	0.571
Primary infertility	4 (8.9)	7 (14.9)		
Secondary infertility	41 (91.1)	40 (85.1)		
Etiology of infertility (*n*, %)			0.235	0.972
Tubal factor	33(73.3)	35 (74.5)		
Ovulatory factor	7 (15.6)	6 (12.8)		
Other	3 (6.7)	4 (8.5)		
Unexplained	2 (4.4)	2 (4.3)		
Basal FSH (IU/L, $\bar{x}\pm s$ )	6.24 ± 1.42	6.53 ± 1.18	−1.103	0.273
Basal LH (IU/L, $\bar{x}\pm s$ )	4.69 ± 0.88	4.55 ± 0.93	0.718	0.474
AMH (ng/mL, $\bar{x}\pm s$ )	3.98 ± 0.59	4.02 ± 0.71	−0.295	0.768
Basal E_2_ (pg/mL, $\bar{x}\pm s$ )	45.26 ± 12.37	46.33 ± 14.02	−0.387	0.699
Number of embryos transferred [*n*, M (Q_45_, Q_75_)]	1.00 (1.00, 1.00)	1.00 (1.00, 1.00)	−1.536	0.124

BMI, body mass index; FSH, follicle-stimulating hormone; LH, luteinizing hormone; AMH, anti-Müllerian hormone; E_2_, estradiol.

### Comparison of endometrial parameters before and after treatment

**Figure 3:**
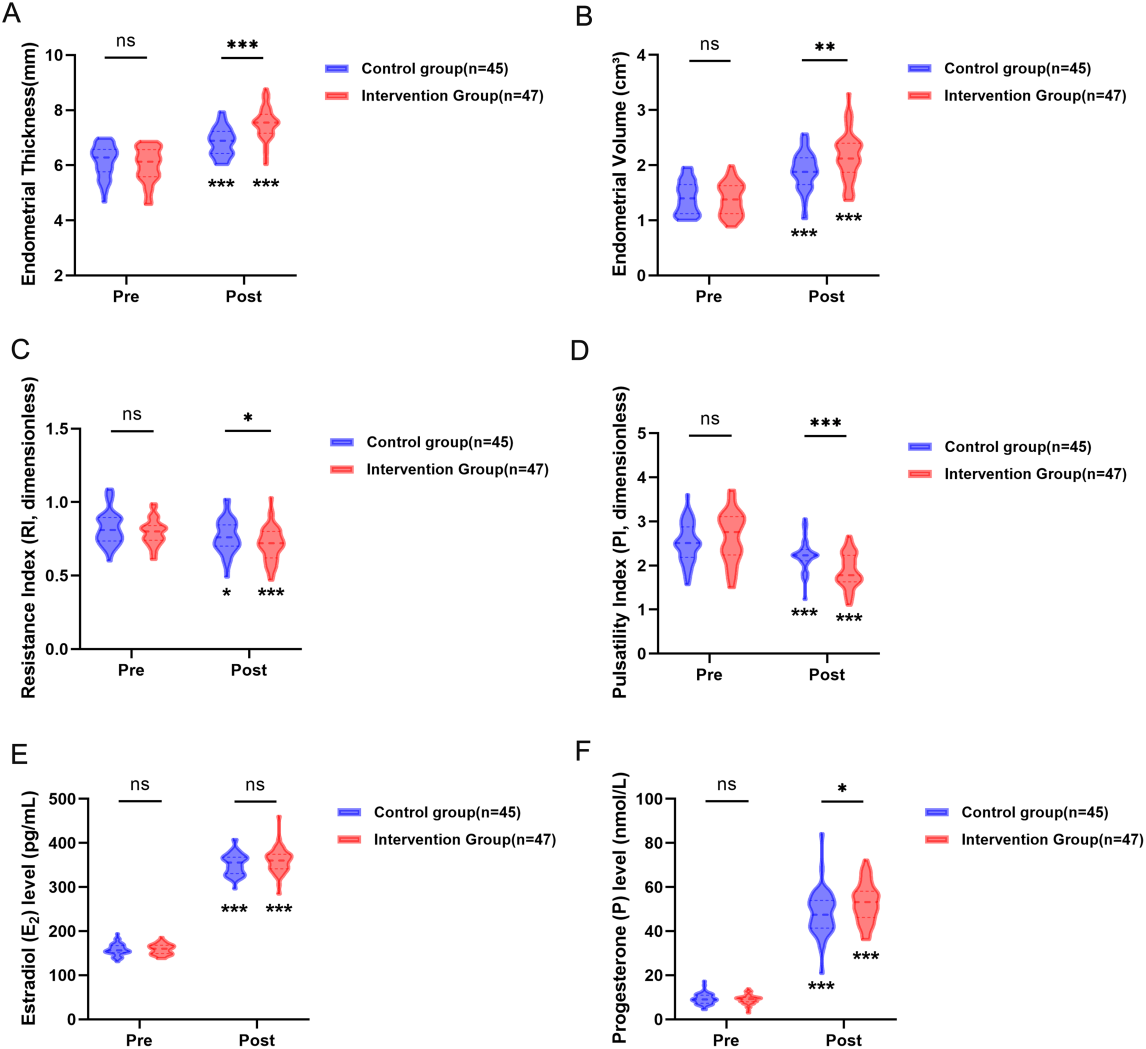
Comparison of endometrial parameters between the two groups before and after treatment. **(A)** Endometrial thickness (mm); **(B)** endometrial volume (cm^3^); **(C)** Resistance index (RI, dimensionless); **(D)** Pulsatility index (PI, dimensionless); **(E)** estradiol (E_2_); **(F)** progesterone (P); ns: *p >* 0.05; ^*^p < 0.05; ^**^p < 0.01; ^***^p < 0.001. Data are presented as mean ± SD.

Changes in endometrial parameters before and after treatment for both groups are shown in Figure 3. At baseline, there were no significant differences between the two groups in endometrial thickness, volume, RI, or PI (all *p* > 0.05). After treatment, both endometrial thickness (Figure 3A) and volume (Figure 3B) were significantly greater in the Intervention Group compared to the control group (both *p* < 0.05). Conversely, the RI and PI values were significantly lower in the Intervention Group (Figures 3C,D, both *p* < 0.05). Intra-group comparison analysis revealed that both groups exhibited a significant increase in endometrial thickness and volume, as well as a significant decrease in RI and PI values after treatment compared to before treatment (all *p* < 0.05).

### Changes in hormone levels before and after treatment

Before treatment, there was no statistically significant difference in E₂ or P levels between the two groups (all *p* > 0.05). After treatment, both E₂ and P levels increased significantly compared to pre-treatment levels in both groups (all *p* < 0.05). Intergroup comparison revealed that E₂ levels remained comparable between the two groups after treatment (*p* > 0.05), whereas P levels in the Intervention group were significantly higher than those in the control group (*p* < 0.05) (Figures 3E,F).

### Comparison of endometrial pattern distribution and subendometrial blood flow grading before and after treatment

**Figure 4:**
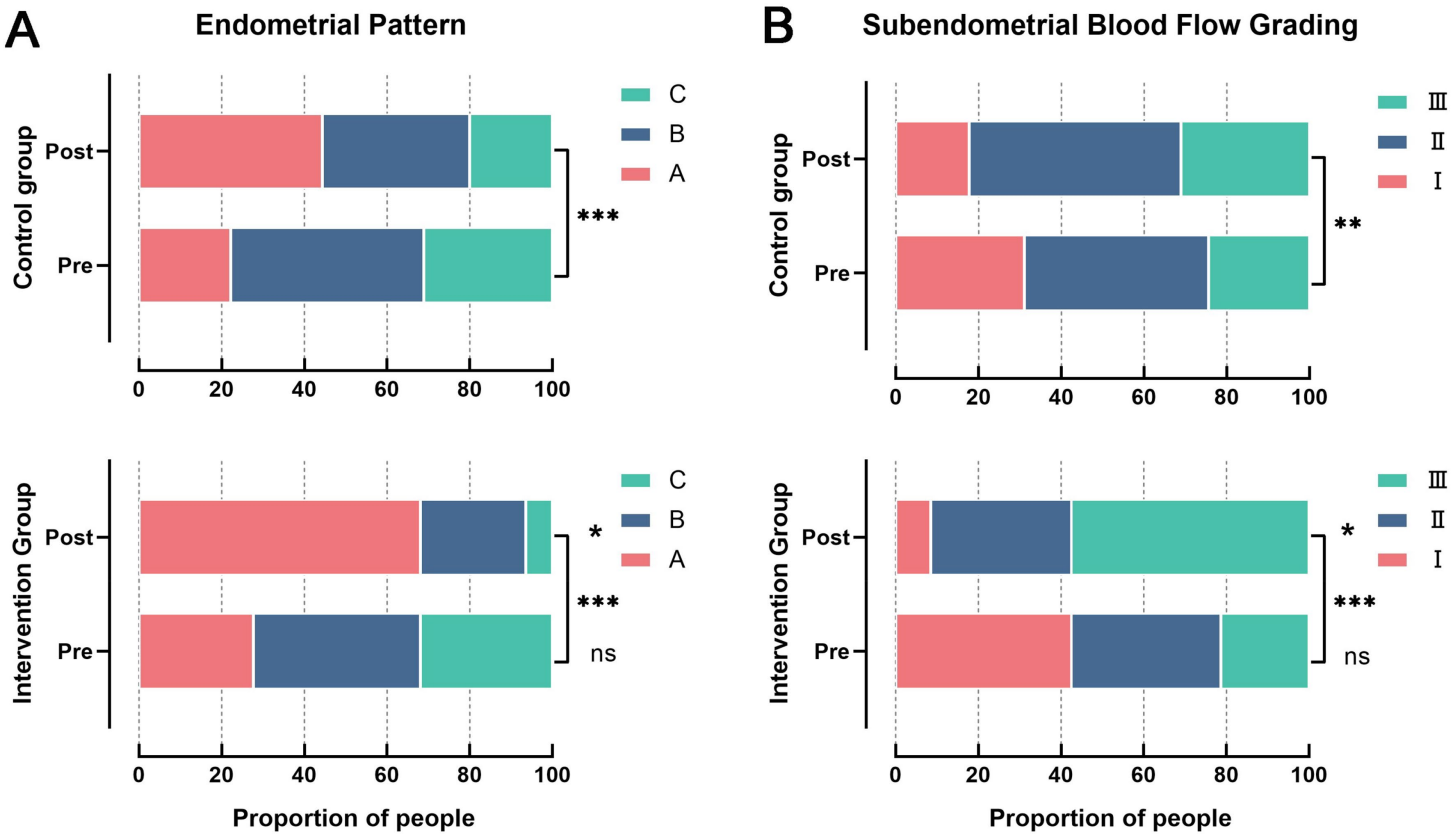
Distribution of endometrial pattern and subendometrial blood flow grading before and after treatment. **(A)** Endometrial pattern; **(B)** subendometrial blood flow grading; ns: *p* > 0.05; ^*^p < 0.05; ^**^p < 0.01; ^***^p < 0.001.

The distribution of endometrial patterns and blood flow grades before and after treatment for both groups is shown in Figure 4. Regarding endometrial patterns (Figure 4A), the Intervention Group showed significant improvement after treatment: the proportion of Pattern C endometrium significantly decreased, while the proportion of Pattern A endometrium significantly increased (both *p* < 0.05), with minimal change in Pattern B proportion. Although the control group showed a similar trend, the magnitude of change was smaller, and the post-treatment improvement was significantly less than that in the Intervention Group (*p* < 0.05). In terms of subendometrial blood flow grading (Figure 4B), the Intervention Group demonstrated significantly improved blood perfusion after treatment, characterized by a significant increase in Grade III flow and a significant decrease in Grade I flow (both *p* < 0.05). The control group showed only minor improvement after treatment, with the degree of blood perfusion improvement being significantly lower than that in the Intervention Group (*p* < 0.05).

### Pregnancy outcome

The pregnancy outcomes for both groups are presented in Table 2. The clinical pregnancy rate in the Intervention Group (55.32%) was significantly higher than that in the control group (31.11%). The embryo implantation rate in the Intervention Group (48.81%) was also significantly higher than that in the control group (32.50%) (both *p* < 0.05). However, there was no statistically significant difference in the early miscarriage rate between the two groups (*p* > 0.05).

**Table 2:** Comparison of pregnancy outcomes between the two groups.

Group	CPR	Implantation rate	Early miscarriage rate
Control group (*n* = 45)	31.11 (14/45)	32.50 (26/80)	14.29 (2/14)
Intervention group (*n* = 47)	55.32 (26.00/47)	48.81 (41/84)	12.50 (3/24)
*χ*^2^	4.541	4.511	0.116
*p*	0.033	0.034	0.736

CPR, clinical pregnancy rate.

## Discussion

This randomized controlled trial investigated the effect of combining Tiao-Jing-Cu-Yun Acupuncture with standard estradiol valerate treatment on improving endometrial receptivity and pregnancy outcomes in infertility patients with thin endometrium undergoing AC-FET. We found that, compared with estrogen therapy alone, the combined acupuncture treatment more significantly increased endometrial thickness and volume, decreased uterine artery RI and PI, optimized endometrial pattern and subendometrial blood flow grading, and ultimately increased the clinical pregnancy rate from 31.11% in the control group to 55.32% and the embryo implantation rate from 32.50 to 48.81% in the Intervention Group (all *p* < 0.05). These results suggest that Tiao-Jing-Cu-Yun Acupuncture, as an adjuvant therapy, can improve the endometrial receptive state through multiple dimensions, thereby creating more favorable conditions for embryo implantation and ultimately enhancing clinical pregnancy success rates in this patient population.

Multiple studies indicate that acupuncture may improve the endometrial environment through mechanisms such as regulating pelvic blood flow, influencing the endocrine axis, and alleviating stress. Research by Dong et al. (16) reported that acupuncture administered during *in vitro* fertilization (IVF) cycles could increase endometrial thickness and improve blood flow parameters, with mechanisms related to the regulation of vasoactive substances and reduction of sympathetic nerve tension. Qi et al. (17) demonstrated that combined intrauterine physical therapy and acupuncture significantly improved endometrial receptivity in patients with thin endometrium by regulating the AMPK/mTOR signaling pathway, thereby enhancing endometrial pattern, blood flow parameters, and the expression of related molecular markers, ultimately increasing embryo implantation and clinical pregnancy rates. This aligns closely with our observation of significantly decreased endometrial blood flow resistance and improved blood perfusion grading in the combined Intervention group, providing direct evidence for acupuncture’s role in improving local uterine microcirculation.

However, controversy remains within the academic community regarding whether acupuncture can definitively increase live birth rates, with conclusions from some high-quality studies being inconsistent. For instance, a 2018 study by Smith et al. (18) published in JAMA did not find a beneficial effect of acupuncture on live births. Nevertheless, several systematic reviews and meta-analyses (19, 20) have highlighted substantial heterogeneity across studies and suggested that the overall effect of acupuncture outcomes is inconclusive, with potential benefits possibly restricted to specific subgroups. The inconsistency across trials may be attributed to differences in study populations, acupuncture protocols (e.g., point selection, treatment timing, and frequency), and sham acupuncture controls.

Importantly, most previous studies enrolled general populations, whereas our trial focused specifically on patients with thin endometrium: a phenotype characterized by poor endometrial receptivity and limited response to conventional estrogen therapy. It is plausible that acupuncture may confer greater benefit in this particular subgroup by improving local uterine blood flow and endometrial angiogenesis, effects that might be less detectable in unselected populations. Therefore, it is premature to dismiss the efficacy of acupuncture for improving live birth rates based solely on negative results from general cohorts. Acupuncture, as a characteristic therapy of traditional medicine, can significantly regulate local blood circulation, improve endometrial receptivity, and alleviate anxiety states, creating favorable conditions for embryo implantation (21). Currently, due to a lack of high-quality, large-sample trials, debate persists over whether acupuncture can truly improve clinical pregnancy rates (CPR) and live birth rates (LBR) in women undergoing IVF (20, 22). This study focused specifically on the challenge of thin endometrium and employed a specific point combination protocol aimed at “tonifying the kidney and spleen, harmonizing the Chong and Ren vessels, and calming the heart and mind.” From a modern medical perspective, this comprehensive protocol may synergistically act on the hypothalamic–pituitary–ovarian (HPO) axis, optimizing the hormonal environment, while simultaneously promoting endometrial angiogenesis and glandular development through local neurohumoral regulation. Therefore, the positive effects we observed may be the integrated result of this targeted protocol’s multi-target intervention on the pathological state of thin endometrium.

Furthermore, the secondary outcome measures in this study provide important intermediary evidence explaining the improvement in clinical pregnancy rates. After treatment, patients in the combined acupuncture group showed a greater tendency for their endometrial pattern to transform into the typical Pattern A triple-line sign, while subendometrial blood flow showed a higher proportion of Grade III signals. Pattern A endometrium and rich subendometrial blood flow are recognized sonographic markers reflecting good endometrial receptivity, signifying synchronized glandular development, adequate stromal edema, and good vascularization (23, 24). The advantage of the Intervention group in these indicators suggests that the benefit of acupuncture may not merely be mechanistically promoting endometrial proliferation but may more importantly improve the structural and functional quality of the endometrium, bringing it closer to the ideal window state for embryo implantation. Notably, the Intervention group exhibited higher P levels. This suggests that Tiao-Jing-Cu-Yun Acupuncture may enhance the efficacy of luteal support by modulating the hypothalamic–pituitary–ovarian axis or improving ovarian and uterine blood flow. The increased P exposure may have further promoted the synchronized secretory transformation of the endometrium. Additionally, the lack of a statistically significant difference in early miscarriage rates between the two groups may imply that acupuncture’s primary contribution lies in overcoming the initial barrier of embryo implantation. Its impact on the maintenance of early pregnancy post-implantation was not apparent in this study’s scale and may require larger sample sizes and longer follow-up to elucidate.

Certainly, this study has several limitations. First, due to the nature of the intervention, blinding of patients and practitioners was not feasible. While common in acupuncture research, this may introduce expectation bias. Additionally, we did not formally test the success of blinding of outcome assessors, which could introduce potential detection bias. Second, this was a single-center study. Although the sample size met the requirements for primary outcome analysis, it remains limited, potentially affecting the generalizability of the results. Third, the assessment of endometrial receptivity relied mainly on sonographic indicators, lacking direct histological or molecular biological evidence, which limits a deeper understanding of the intrinsic molecular mechanisms of acupuncture’s action. Fourth, the main result of this study is the clinical pregnancy rate, which is an intermediate surrogate indicator. The testing efficacy of this study is insufficient to detect the difference in live birth rate, while the live birth rate remains the gold standard outcome in reproductive medicine trials. Fifth, several secondary ultrasound and hormonal parameters were compared without adjustment for multiple comparisons. Therefore, these secondary findings should be interpreted as exploratory and hypothesis-generating. However, this study preliminarily indicates that for patients with thin endometrium, adjuvant Tiao-Jing-Cu-Yun Acupuncture during hormone replacement therapy (HRT) cycles can effectively improve endometrial receptivity parameters and increase clinical pregnancy and embryo implantation rates, offering a new therapeutic approach for this clinical challenge. Future validation through multicenter, large-sample studies with rigorous controls is needed, along with deeper exploration of the underlying biological pathways.

## Data Availability

The raw data supporting the conclusions of this article will be made available by the authors, without undue reservation.
